# Endocannabinoid system modulation in bruxism: a neurobiological hypothesis and translational model of ECS-targeted intervention

**DOI:** 10.3389/fnins.2026.1854001

**Published:** 2026-07-01

**Authors:** Rafaela A. da Rosa, Ricardo Armini Caldas

**Affiliations:** Department of Dentistry, Federal University of Santa Catarina, Florianópolis, Brazil

**Keywords:** bruxism, cannabinoids, endocannabinoid system, motor control, neuromodulation, orofacial pain

## Abstract

Bruxism is a multifactorial motor behavior of predominantly central origin, characterized by repetitive masticatory muscle activity and associated with dysregulation of dopaminergic, serotonergic, GABAergic, and glutamatergic pathways involved in motor control, emotional regulation, and stress responsivity. The endocannabinoid system (ECS) has emerged as a key homeostatic neuromodulator capable of integrating these neurotransmitter systems, thereby influencing pain processing, sleep–wake dynamics, and motor output. This article develops a neurobiological hypothesis based on a narrative integrative synthesis of clinical, experimental, and translational evidence regarding ECS involvement in the pathophysiology of bruxism. Findings from randomized clinical trials suggest that topical cannabidiol (CBD) may modulate motor neuron excitability and reduce pain-related outcomes, while case-based and experimental evidence supports the interaction between cannabinoid signaling and neural circuits implicated in motor control and behavioral regulation. Building on this evidence, we propose a hypothesis-driven translational model in which ECS-mediated neuromodulation may influence central mechanisms underlying bruxism, including motor pattern generation, stress responsivity, and nociceptive processing. Rather than providing prescriptive therapeutic recommendations, this model is intended as a hypothesis-generating construct that integrates current knowledge on ECS signaling within the broader neurobiology of motor control. Although heterogeneity in study design and outcome measures limits definitive conclusions, the available evidence supports the ECS as a plausible modulatory system in bruxism, with potential implications for future mechanistic and clinical research in centrally mediated motor disorders.

## 1 Introduction

Bruxism is currently understood as a multifactorial motor behavior characterized by repetitive masticatory muscle activity during sleep and/or wakefulness, which may act as a risk factor, a protective factor, or a neutral behavior depending on the clinical context (Verhoeff et al., 2025). Its expression involves central mechanisms related to motor control, stress responsivity, and emotional regulation, with dopaminergic, GABAergic, serotonergic, and glutamatergic pathways participating at different levels of modulation. This integrative review examines the role of the endocannabinoid system (ECS) in the pathophysiology and modulation of these central mechanisms involved in bruxism.

From a clinical standpoint, bruxism is defined as a repetitive masticatory muscle activity characterized by clenching or grinding of the teeth and/or by mandibular thrusting, and it can manifest during sleep (sleep bruxism) or wakefulness (awake bruxism) (Verhoeff et al., 2025). Sleep bruxism corresponds to the occurrence of masticatory muscle activity during sleep, with rhythmic (phasic) or non-rhythmic (tonic) episodes, and is currently understood as a sleep-related behavioral manifestation rather than a movement or sleep disorder. In turn, awake bruxism refers to an increase in masticatory muscle activity in the awake state, characterized by repetitive or sustained tooth contact and/or by mandibular thrusting, and is also not classified as a movement disorder (Verhoeff et al., 2025).

Although they share phenotypic similarities, sleep bruxism and awake bruxism are distinct entities in terms of pathophysiology, neural modulation, and clinical implications. Historically, the management of bruxism has focused on containing its peripheral effects—such as tooth wear, orofacial pain, and joint overload—through the use of interocclusal devices and musculoskeletal therapies. However, advances in neuroscience have shown that bruxism has a predominantly central origin, involving the interaction of dysfunctions in the dopaminergic, GABAergic, serotonergic, and glutamatergic systems, which play a fundamental role in motor control, emotional regulation, and reward mechanisms (Lavigne et al., 2003; Manfredini and Lobbezoo, 2010; Manfredini et al., 2013).

The COVID-19 pandemic highlighted the sensitivity of bruxism to psychosocial stressors, emotional changes, and contextual factors. Studies conducted in different populations reported increases in oral parafunctions, tooth clenching, tooth grinding, orofacial pain, and temporomandibular disorder symptoms during periods of social isolation, health-related uncertainty, and occupational instability, especially in individuals exposed to higher levels of stress, anxiety, and psychoemotional distress (Emodi-Perlman et al., 2020; Saccomanno et al., 2020; Cullen et al., 2020; Emodi-Perlman and Eli, 2021; Badaró et al., 2021; Rocha et al., 2021; da Silva et al., 2021; Winocur-Arias et al., 2022; Generoso et al., 2022). These findings do not indicate a single causal mechanism, but reinforce the understanding of bruxism as a motor behavior sensitive to the interaction between stress, emotional regulation, sleep, pain, and environmental context.

In this context, the endocannabinoid system (ECS) has come to be considered a relevant neuromodulatory target, given its broad participation in the modulation of pain, inflammation, the stress response, motor control, and sleep–wake cycles (Di Marzo and Piscitelli, 2015; Covey et al., 2017; Di Marzo, 2018). Structurally, the ECS comprises cannabinoid receptors (CB1 and CB2), endogenous ligands such as anandamide and 2-arachidonoylglycerol (2-AG), as well as the enzymes responsible for their synthesis and degradation. This system interacts directly with neurotransmitter circuits involved in the genesis and modulation of bruxism, reinforcing its importance for the neurobiological understanding of the disorder (Covey et al., 2017; Di Marzo, 2018).

The conceptual evolution of the field has led to the proposition of the endocannabinoidome, which expands the understanding of the ECS to include other lipid mediators, such as palmitoylethanolamide (PEA) and oleoylethanolamide (OEA), as well as additional receptors, including TRPV1, peroxisome proliferator-activated receptors (PPARs), and GPR55. This integrated set acts in a coordinated manner on neuroendocrine, immunological, and neuromuscular axes, influencing complex physiological processes related to the stress response, nociception, and muscle tone (Kogan and Mechoulam, 2007; Di Marzo, 2018; Cristino et al., 2020; Iannotti and Di Marzo, 2025).

From this expanded perspective, phytocannabinoids—including cannabidiol (CBD), tetrahydrocannabinol (THC), cannabigerol (CBG), and cannabinol (CBN)—have been gaining prominence for their neuromodulatory potential in different conditions, such as neuromotor disorders, chronic pain, anxiety disorders, and sleep disturbances (Fernández-Ruiz et al., 2015; Whiting et al., 2015; Shannon et al., 2019; Hakami and Alshehri, 2025; Perez et al., 2025). Within the dental and orofacial neuroscience context, although the clinical application of this field is still emerging, recent evidence suggests that ECS modulation can reduce involuntary muscle activity, improve sleep quality, and attenuate emotional states frequently associated with bruxism (Nitecka-Buchta et al., 2019; Rosa and Martins, 2024; Rosa et al., 2025; Walczyńska-Dragon et al., 2024, 2025a, 2025b).

Experimental and clinical evidence indicates that bruxism is closely associated with the activation of stress responses at different physiological levels. Classical studies have demonstrated increased sympathetic activity in individuals with bruxism, through the elevation of urinary catecholamines, suggesting the involvement of the autonomic axis in the expression of parafunctional masticatory activity (Clark et al., 1980; Seraidarian, 2006). In parallel, clinical investigations have observed a significant association between bruxism, psychological stress, anxiety, and a negative impact on quality of life, reinforcing the role of emotional factors as modulators of the condition (Molina, 2006; Abekura et al., 2011; Molina et al., 2011). In experimental models, animal studies have shown that exposure to stress is capable of altering masticatory patterns and inducing behaviors compatible with bruxism, providing support for the hypothesis of central control of this phenomenon (Gómez et al., 1999, 2010).

These findings have contributed to the consolidation of the contemporary understanding of bruxism as a condition of predominantly central origin, refuting the hypothesis of occlusal interferences as a cause of bruxism and reinforcing the need for integrated diagnostic and therapeutic approaches (Manfredini and Lobbezoo, 2009; Manfredini et al., 2020).

Given this complex network of neurotransmitters and circuits involved in the genesis of bruxism, the need to understand neurobiological systems capable of simultaneously integrating, modulating, and regulating multiple central and peripheral pathways becomes evident. In this context, the endocannabinoid system (ECS) emerges as a fundamental homeostatic neuromodulator, with a cross-cutting role across the dopaminergic, GABAergic, serotonergic, and glutamatergic systems, in addition to playing a relevant role in the regulation of stress, pain, sleep, and motor control, providing a unifying neurobiological framework for understanding bruxism as a centrally mediated motor behavior. Understanding the structure, mechanisms of action, and physiological functions of the ECS is, therefore, an essential step to substantiate its neuromodulatory applicability in the management of bruxism.

## 2 Methods

This article is a narrative integrative review with theoretical synthesis, aimed at developing a neurobiological hypothesis regarding the role of the endocannabinoid system (ECS) in the pathophysiology and modulation of bruxism. Considering the scarcity of studies directly evaluating cannabinoid-based interventions in sleep bruxism or awake bruxism, this review integrated evidence directly related to bruxism with studies addressing neurobiological mechanisms and frequently overlapping clinical outcomes, such as masticatory muscle activity, orofacial pain, temporomandibular disorders, sleep, stress, inflammation, neurotransmission, and motor control. This approach was adopted to support a hypothesis-generating neurobiological model, without treating these conditions or outcomes as equivalent to bruxism.

### 2.1 Search strategy and information sources

The literature search was conducted in the PubMed/MEDLINE, Scopus, and Web of Science databases, covering publications from 1980 to 2025. Search terms included combinations of controlled descriptors and free-text terms related to bruxism, sleep bruxism, awake bruxism, masticatory muscle activity, endocannabinoid system, endocannabinoidome, cannabinoids, phytocannabinoids, cannabidiol, tetrahydrocannabinol (Δ9-THC), cannabigerol (CBG), orofacial pain, temporomandibular disorders, CB1 and CB2 receptors, TRPV1, PPARs, GPR55, neurotransmitters, motor control, sleep, stress, inflammation, and neuromodulation. Boolean operators (AND/OR) were applied to refine the search. Examples of search combinations included: (“bruxism” OR “sleep bruxism” OR “awake bruxism”) AND (“endocannabinoid system” OR “cannabinoids” OR “cannabidiol” OR “CBD”); (“bruxism” OR “masticatory muscle activity”) AND (“dopamine” OR “GABA” OR “glutamate” OR “serotonin”); and (“temporomandibular disorder” OR “orofacial pain” OR “myofascial pain”) AND (“cannabidiol” OR “cannabinoids” OR “endocannabinoid system”). Additionally, manual screening of reference lists from selected studies was performed to identify relevant articles not retrieved through database searches.

### 2.2 Eligibility criteria

Studies were considered eligible if they addressed the relationship between the endocannabinoid system and/or endocannabinoidome, bruxism, and associated neurobiological mechanisms, or if they investigated cannabinoid-based interventions in relevant clinical or experimental contexts. Studies on orofacial pain, temporomandibular disorders, electromyographic masticatory activity, sleep, stress, inflammation, neurotransmission, and motor control were also included when these topics contributed to the interpretation of mechanisms or outcomes related to the proposed model. Included study designs comprised randomized and non-randomized clinical trials, observational studies, experimental studies in animal models, mechanistic studies, systematic and narrative reviews, integrative reviews, hypothesis or theory articles, and case reports.

Publications without full-text availability, duplicate records, and studies not directly related to the objectives of this article were excluded. Exclusively epidemiological or clinical studies without neurobiological, pharmacological, or interpretative contribution to the proposed model were used only when necessary for contextualization.

### 2.3 Study selection and data extraction

Study selection was conducted through sequential screening of titles, abstracts, and full texts. Relevant data were extracted regarding study design, population or experimental model, clinical condition evaluated, type of cannabinoid intervention, including formulation, dose, and route of administration when applicable, outcomes evaluated, such as orofacial pain, electromyographic activity, sleep parameters, and behavioral measures, main findings, and methodological limitations.

In addition, the included studies were critically classified according to type of evidence, direct or indirect relationship with bruxism, clinical condition evaluated, primary outcome, bruxism-related outcomes, and main methodological limitations. This classification was used to construct Table 1 and to differentiate direct evidence on bruxism from indirect evidence derived from studies on orofacial pain, temporomandibular disorders, masticatory muscle activity, sleep, or overlapping clinical conditions.

**Table 1:** Critical synthesis of clinical studies evaluating cannabinoid-based interventions in bruxism and related orofacial conditions.

Study	Study design	Population/condition evaluated	Cannabinoid intervention and route	Main outcomes	Findings relevant to bruxism or masticatory muscle activity	Relationship of the evidence with bruxism	Main limitations
Nitecka-Buchta et al. (2019)	Randomized, double-blind clinical trial	Individuals with myofascial pain related to temporomandibular disorders	CBD formulation applied transdermally over the masseter muscle	Pain intensity (VAS) and masseter muscle activity (sEMG)	Reduction in masseter electromyographic activity and pain intensity compared with placebo	Indirect evidence: relevant to masticatory muscle activity and myofascial pain, but bruxism was not the primary endpoint	Short follow-up; TMD/myofascial pain population; absence of SB/AB as a direct primary endpoint
Walczyńska-Dragon et al. (2024)	Randomized, double-blind, three-arm clinical trial	Adults with sleep bruxism and muscle-related TMD	CBD 10%, CBD 5%, or placebo applied to the masseter region	Pain intensity, sEMG activity, and sleep bruxism intensity assessed by Bruxoff	Reduction in pain, sEMG activity, and sleep bruxism intensity compared with placebo	Direct clinical evidence for sleep bruxism associated with muscle-related TMD	Short follow-up; combined bruxism, pain, and TMD outcomes; limited sample size
Walczyńska-Dragon et al. (2025a)	Randomized, double-blind clinical trial	Individuals with muscular pain associated with bruxism	CBD 5%, CBD 10%, or placebo gel applied intraorally to the masseter muscles	Sleep quality (PSQI), migraine-related disability (MIDAS), sEMG, and Bruxoff	Improvement in sleep quality and migraine-related disability; sEMG findings supported reduced muscle tension, with correlation between clinical improvement, reduced muscle activity, and pain	Associated evidence: evaluates comorbid outcomes in muscular pain associated with bruxism, with objective assessment of muscle tension and bruxism intensity	Short follow-up; associated outcomes rather than isolated SB/AB as primary endpoints; absence of pharmacokinetic assessment
Pina-Escudero et al. (2021)	Case report	Awake bruxism in behavioral variant frontotemporal degeneration	Oral CBD capsule	Bruxism severity and clinical course	Awake bruxism was almost completely relieved after CBD use	Direct evidence for awake bruxism, but limited to a single neurological case	Single case; absence of control group; specific neurodegenerative condition; limited generalizability
Tanganeli et al. (2023)	Case report	Chronic temporomandibular arthralgia	Full-spectrum cannabidiol formulation	Pain and mandibular function	Reduction in pain and functional improvement in a painful TMD-related condition	Indirect evidence: relevant to orofacial pain and TMD-related function, not direct evidence for SB/AB	Single case; absence of control group; bruxism not established as a primary outcome
Pinheiro et al. (2025)	Case report	Chronic myofascial temporomandibular disorder with sleep dysfunction	Full-spectrum Cannabis oil	Pain intensity and sleep quality	Improvement in orofacial pain and sleep quality	Indirect evidence: relevant to pain and sleep dysfunction in myofascial TMD, not direct evidence for SB/AB	Single case; absence of control group; absence of objective bruxism outcome
Walczyńska-Dragon et al. (2025b)	Narrative review	Temporomandibular disorders and orofacial pain	CBD-based interventions reviewed across preclinical and clinical studies	Pain, inflammation, muscle hyperactivity, routes of administration, and safety	Supports the biological plausibility of CBD in TMD-related pain, inflammation, and muscle hyperactivity	Contextual evidence: supports mechanistic and clinical rationale, but does not provide original bruxism intervention data	Review-level evidence; high heterogeneity of included studies; absence of meta-analysis

AB, awake bruxism; CBD, cannabidiol; MIDAS, Migraine Disability Assessment; PSQI, Pittsburgh Sleep Quality Index; SB, sleep bruxism; sEMG, surface electromyography; TMD, temporomandibular disorder; VAS, visual analogue scale.

### 2.4 Data synthesis

Due to the heterogeneity of study designs, populations, cannabinoid formulations, routes of administration, diagnostic criteria, and outcome measures, a qualitative narrative synthesis was performed. Findings were organized into thematic domains, including: (i) neurobiological mechanisms and modulators of bruxism; (ii) interactions between the ECS and neurotransmitter systems involved in motor control and nociception; (iii) clinical and experimental evidence of cannabinoid use; and (iv) development of a hypothesis-driven translational model integrating ECS modulation within the neurobiology of bruxism.

## 3 Integrative literature review and translational discussion

### 3.1 Mechanisms and multifactorial nature of bruxism

Bruxism is currently understood as a motor behavior of predominantly central origin, characterized by repetitive activity of the masticatory muscles, whose clinical expression results from the dynamic interaction between primary neurobiological mechanisms and multiple associated modulatory factors. This conception reflects the consolidation of clinical, experimental, and epidemiological evidence that has definitively moved away from historically proposed peripheral hypotheses, particularly those based on isolated occlusal interferences, which lack consistent scientific support and have been progressively refuted by contemporary literature (Lavigne et al., 2008; Lobbezoo et al., 2008; Manfredini et al., 2015; Conti et al., 2024).

According to the recently updated International Consensus on Bruxism, bruxism should be classified according to the circadian cycle into sleep bruxism (SB) and awake bruxism (AB), recognizing that, although they share a common central basis, these two manifestations present partially distinct pathophysiological, contextual, and behavioral modulators (Manfredini et al., 2022; Verhoeff et al., 2025). This distinction is fundamental for understanding the etiology, clinical interpretation, and therapeutic approach to bruxism.

Classical and contemporary evidence demonstrates that bruxism is consistently associated with the activation of stress responses at different physiological levels. Pioneering studies identified increased sympathetic activity in individuals with bruxism, evidenced by elevated levels of urinary catecholamines, suggesting direct involvement of the autonomic axis in the genesis of parafunctional masticatory activity (Clark et al., 1980; Seraidarian, 2006). Subsequent investigations reinforced this association by demonstrating a significant correlation between bruxism, psychological stress, anxiety, and negative impacts on quality of life (Molina, 2006; Abekura et al., 2011; Molina et al., 2011).

Experimental models provided mechanistic support for this central hypothesis. In animal studies, exposure to psychological stressors was capable of inducing central dopaminergic alterations and an increase in non-functional masticatory activities, compatible with stereotyped patterns observed clinically in bruxism, reinforcing the role of stress as a trigger and modulator of orofacial motor expression (Gómez et al., 1999, 2010).

In the specific context of sleep bruxism, the literature demonstrates that episodes of masticatory muscle activity occur predominantly during light sleep stages (N1 and N2), frequently in temporal association with microarousals and autonomic activation, and in a lesser proportion during REM sleep. These findings support the interpretation of SB as a motor phenomenon related to cortical arousal mechanisms and sleep instability, rather than as a primary movement disorder or exclusively respiratory condition (Lavigne et al., 2003; Saito et al., 2016; Tsujisaka et al., 2018).

Beyond the primary central mechanisms, contemporary literature recognizes the existence of secondary factors associated with bruxism, capable of initiating, maintaining, or exacerbating parafunctional activity, both in sleep bruxism and awake bruxism. These factors do not constitute primary causes, but rather act as modulators of central excitability, sleep–wake cycle architecture, and autonomic activation, contributing to the clinical variability observed among individuals. Among the most widely investigated secondary factors are sleep-related breathing disorders, gastrointestinal conditions, psychoactive substance use, as well as certain neurological and pharmacological conditions (Rosa and Martins, 2024).

In this context, sleep-related breathing disorders have been widely investigated as secondary factors associated with sleep bruxism. Polysomnographic studies indicate a high prevalence of SB in individuals with obstructive sleep apnea (OSA), with a significant portion of masticatory muscle activity episodes occurring in temporal proximity to microarousal events, although without consistent association with specific apneas or hypopneas (Saito et al., 2016; Tsujisaka et al., 2018). Systematic and integrative reviews point to high methodological heterogeneity and the absence of a direct causal relationship between OSA and SB, suggesting that both conditions share central mechanisms, such as sleep fragmentation and autonomic activation, acting as contextual modulators (Jokubauskas and Baltrušaitytė, 2017; Ferreira et al., 2023).

From a clinical perspective, this interface assumes special relevance in the indication of interocclusal devices. Evidence demonstrates that in individuals with previously diagnosed OSA, the use of stabilizing splints can worsen nocturnal breathing disorders, increasing the apnea-hypopnea index, the frequency of microarousals, and snoring time, reinforcing the need for respiratory screening before their routine prescription (Gagnon et al., 2004).

Furthermore, gastrointestinal conditions, particularly gastroesophageal reflux, also figure among the secondary factors associated with sleep bruxism. Clinical studies indicate a significant increase in the prevalence of SB in individuals with reflux, possibly mediated by mechanisms of microarousal and nocturnal autonomic activation. The mandibular movement observed during bruxism episodes may exert an adaptive effect by increasing salivary flow and contributing to acid buffering, although no direct causal relationship is established between these conditions (Mengatto et al., 2013).

Complementarily, contemporary literature recognizes that bruxism is strongly influenced by other associated secondary factors, capable of modulating its clinical expression according to individual phenotype. Among these factors, the consumption of licit psychoactive substances stands out as a widely documented axis. Cohort and twin studies demonstrate a robust association between bruxism and nicotine dependence, with an increased risk of 1.5 to 2.9 times in smokers, in addition to a dose–response relationship according to the number of cigarettes consumed (Rintakoski et al., 2010).

Regular alcohol consumption also shows a moderate association with sleep bruxism, possibly mediated by an increase in respiratory events, sleep fragmentation, and autonomic activation (Rintakoski and Kaprio, 2013; Simou et al., 2018). Caffeine, in turn, has been implicated as a relevant secondary modulatory factor, especially when consumed in high quantities, being associated with an increase in masticatory activity during wakefulness and states of greater central excitability, possibly mediated by its stimulant effects on the central nervous system and by interference with sleep architecture (Quadri et al., 2015).

The use of psychostimulants and psychotropic medications constitutes another important group of associated factors. Antidepressants, especially selective serotonin and norepinephrine reuptake inhibitors, as well as methylphenidate, have been consistently related to the induction or exacerbation of bruxism, both sleep and awake, possibly by interfering with the inhibitory dopaminergic balance and excitatory serotonergic modulation on trigeminal motor nuclei (Falisi et al., 2014; Sivri and Bilgiç, 2015; Uca et al., 2015).

Neurological and psychiatric conditions also figure as relevant associated factors, including cerebral palsy, Parkinson’s disease, psychiatric disorders, and substance use disorders, in which a higher prevalence of oral parafunctional habits and clinical signs of bruxism are observed, reinforcing the relationship between central dysfunctions, motor control, and masticatory behavior (Ortega et al., 2007; Winocur et al., 2007; Verhoeff et al., 2018).

Recent systematic and narrative reviews consolidate this panorama by demonstrating that bruxism results from the dynamic overlap of primary and secondary factors, whose relative contribution varies according to age, psychosocial context, sleep quality, clinical comorbidities, and substance use. In adolescents and young adults, psychosocial and behavioral factors exert predominant influence, while in specific clinical populations, the pharmacological and neurobiological contribution becomes more evident (Castroflorio et al., 2017a; Conti et al., 2024).

Recent studies have also broadened the understanding of bruxism by integrating etiological factors, behavioral patterns, and more sensitive measurement tools. Systematic reviews in adults confirm that bruxism results from the interaction between psychosocial factors, sleep disorders, substance use, pain, and behavioral variables, reinforcing its multifactorial nature and the absence of a single causal axis (Castroflorio et al., 2017b). In the context of awake bruxism, electromyographic investigations demonstrate that individuals with myofascial pain present a higher frequency of tooth clenching episodes during standardized concentration tasks, when compared to asymptomatic controls, evidencing the influence of nociceptive state and cognitive context on masticatory activity (Cioffi et al., 2017). Technological advances, such as ultrasmall wearable EMG systems, have enabled continuous recording of masseter activity throughout the day, expanding the ecological characterization of individual muscle contraction patterns (Yamaguchi et al., 2018). Furthermore, interventions based on electromyographic biofeedback during wakefulness have demonstrated the capacity to modulate the phasic component of sleep bruxism, reinforcing the functional interconnection between SB and AB and the role of behavioral self-regulation in the modulation of central motor excitability (Saito-Murakami et al., 2020).

Given this complexity, bruxism should be understood as a manifestation of multifactorial neuromodulatory dysregulation, sensitive to the interaction between primary central mechanisms and associated secondary factors, and not as an isolated entity or one that can be explained by a single mechanism. This understanding supports the need for integrative explanatory models capable of simultaneously encompassing motor control, stress response, sleep architecture, and pain modulation. In this context, the analysis of the main neurotransmitter pathways involved in bruxism reveals a fundamental point of convergence: the cross-cutting action of the endocannabinoid system, and of its expanded system, the endocannabinoidome, as a key modulator of these circuits, providing the biological rationale for its therapeutic exploration, discussed in the following section.

### 3.2 Neurotransmitters and pathways involved in masticatory motor control

The neuromotor control of the masticatory muscles depends on the integration of the motor cortex, basal ganglia, thalamus, brainstem, and limbic system, with participation of dopaminergic, GABAergic, glutamatergic, and serotonergic pathways (DeLong and Wichmann, 2010; Aires, 2015). In bruxism, these pathways should not be interpreted as equivalent causal mechanisms, but as systems that modulate, at different levels, motor control, stress response, neuronal excitability, sleep, pain, and emotional regulation. The endocannabinoid system (ECS) participates in this regulation mainly through retrograde signaling: endocannabinoids produced on demand in the postsynaptic neuron, especially anandamide and 2-AG, act on presynaptic receptors, particularly CB1, modulating the release of neurotransmitters such as GABA and glutamate. Thus, the ECS does not act as a linear blocker or stimulator of these circuits, but as a homeostatic system capable of adjusting synaptic activity according to the functional state of the circuit involved (Di Marzo and Piscitelli, 2015; Covey et al., 2017; Arturo and Fabiana, 2018; Di Marzo, 2018; Iannotti and Di Marzo, 2025).

The dopaminergic pathway is particularly relevant to bruxism because of its relationship with motor control, stress response, reward, and repetitive behaviors. Alterations in dopaminergic neurotransmission are associated with repetitive motor patterns and states of motor hyperactivity, especially in corticostriatal, nigrostriatal, and mesocorticolimbic circuits (Kogan and Mechoulam, 2007; Fernández-Ruiz et al., 2015; Covey et al., 2017; Laksmidewi and Soejitno, 2021). In experimental models, exposure to stress has been associated with changes in central dopaminergic metabolism and increased non-functional masticatory activities, reinforcing the relationship between stress, dopamine, and orofacial motor expression (Gómez et al., 1999, 2010). The ECS can modulate this axis directly and indirectly, especially by controlling GABAergic and glutamatergic inputs that influence dopaminergic neurons. Therefore, endocannabinoid modulation should not be understood simply as increasing or decreasing dopamine, but as adjusting the functional balance between excitation, inhibition, stress response, and motor selection (Covey et al., 2017; Laksmidewi and Soejitno, 2021).

The GABAergic and glutamatergic pathways constitute the main axis controlling neuronal excitability. GABA limits excessive neuronal firing and contributes to the control of muscle tone, whereas glutamate participates in the activation of motor circuits and synaptic plasticity. The ECS regulates this balance through CB1 receptors located on GABAergic and glutamatergic presynaptic terminals. When activated by endocannabinoids, these receptors reduce the release of GABA or glutamate, producing distinct effects depending on the circuit involved: reduced glutamate release may limit neuronal excitation, whereas reduced GABA release may generate disinhibition of specific circuits. This mechanism explains why ECS modulation may contribute to the functional balance of motor circuits without acting as a simple agonist or antagonist of a single neurotransmitter pathway. In the context of bruxism, this logic supports a hypothesis of modulation of motor excitability and masticatory muscle activity, which still needs to be directly tested in mechanistic and clinical studies (Kano et al., 2009; Romero and Martinez-Orgado, 2009; Gerfen and Surmeier, 2011; Katona and Freund, 2012; Di Marzo, 2018; Iannotti and Di Marzo, 2025).

The serotonergic pathway participates in emotional regulation, anxiety, sleep, stress response, and functional interaction with dopaminergic circuits. Clinical evidence indicates that psychotropic medications that increase synaptic serotonin availability, especially selective serotonin reuptake inhibitors, may induce or exacerbate manifestations of bruxism during sleep and wakefulness, possibly through indirect interference with dopaminergic pathways involved in orofacial motor control (Falisi et al., 2014; Uca et al., 2015; Garrett and Hawley, 2018; Manfredini et al., 2020; Wallem et al., 2025). Endocannabinoid signaling also modulates the serotonergic system, especially in circuits involving the dorsal raphe, where CB1 receptors can regulate GABAergic and glutamatergic afferents to serotonergic neurons. This mechanism allows the ECS to influence the excitability of serotonergic neurons and, consequently, responses related to stress, anxiety, and sleep (Haj-Dahmane and Shen, 2011). Thus, in bruxism, serotonin should be understood less as an isolated motor pathway and more as a modulator of secondary factors that may indirectly influence masticatory activity.

In addition to central modulation mediated mainly by CB1, the CB2 receptor becomes relevant in contexts in which bruxism is associated with pain, inflammation, musculoskeletal overload, or peripheral sensitization. CB2-related pathways participate in neuroimmune and inflammatory modulation and may influence orofacial pain, tissue response, and peripheral processes associated with temporomandibular disorders and muscle hyperactivity. Thus, while CB1 is more directly related to central synaptic modulation and neurotransmitter control, CB2 contributes to the anti-inflammatory, analgesic, and immunomodulatory rationale for cannabinoid modulation in phenotypes in which bruxism, myofascial pain, and inflammation coexist (Di Marzo and Piscitelli, 2015; Di Marzo, 2018; Iannotti and Di Marzo, 2025).

This characteristic differentiates ECS modulation from conventional pharmacological interventions that act predominantly on a single receptor, enzyme, or neurotransmitter. Because it acts as a homeostatic system, the ECS may contribute to the functional rebalancing of motor, emotional, nociceptive, autonomic, and sleep-related circuits, without assuming that all individuals with bruxism present the same direction of neurochemical alteration. This point is particularly important because secondary factors associated with bruxism, such as anxiety, sleep disturbances, chronic pain, use of psychostimulants, antidepressants, alcohol, nicotine, and other substances, may modulate the clinical expression of the behavior. In this context, ECS-targeted strategies can be investigated not only for their potential effect on masticatory muscle activity, but also for the possibility of assisting in the management of associated factors, in the review of medications potentially inducing bruxism, and, when clinically indicated, in the gradual reduction or therapeutic substitution of medications under professional supervision.

The endocannabinoidome expands this rationale by including, in addition to CB1, CB2, anandamide, and 2-AG, other lipid mediators, metabolic enzymes, and molecular targets, such as TRPV1, PPARs, GPR55, and ion channels (Arturo and Fabiana, 2018; Iannotti and Di Marzo, 2025). This expanded network helps explain why cannabinoid modulation may simultaneously affect pain, inflammation, sleep, anxiety, stress response, and motor control. Complementarily, cannabidiol has a polypharmacological profile, with multiple molecular targets, which reinforces the plausibility of its investigation in multifactorial conditions in which these domains overlap (Castillo-Arellano et al., 2023).

In summary, dopamine is more directly related to motor control, stress, and repetitive behaviors; GABA and glutamate regulate the excitatory-inhibitory balance and neuronal excitability; serotonin participates in emotional regulation, sleep, and stress response; and CB2 adds the neuroimmune, inflammatory, and analgesic axis to the model. The cross-cutting action of the ECS on these systems supports a plausible neurobiological rationale for investigating its modulation in bruxism, especially in phenotypes in which masticatory muscle activity, pain, sleep, anxiety, stress, and pharmacological factors overlap. This interpretation remains hypothetical and translational, and should not be read as direct evidence of causality between the ECS and all clinical phenotypes of bruxism.

**Figure 1:**
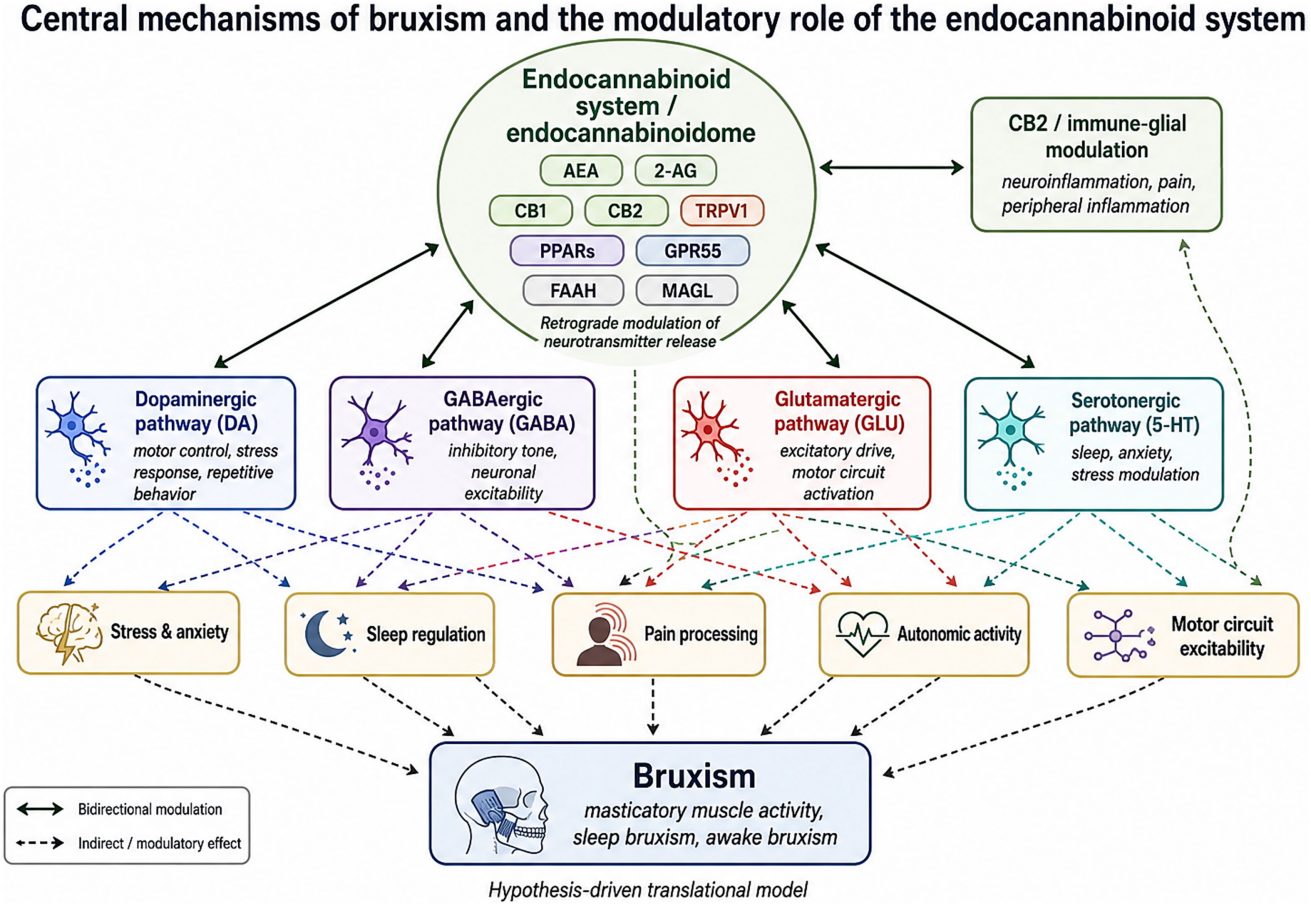
Integrative model of central modulation of bruxism by the endocannabinoid system. The diagram represents the interaction between the ECS/endocannabinoidome, neurotransmitter pathways, immune-glial modulation, and functional domains involved in the clinical expression of bruxism, including motor circuit excitability, stress and anxiety, sleep regulation, pain processing, and autonomic activity. Solid arrows indicate bidirectional modulation, whereas dashed arrows indicate indirect or modulatory effects. The model is hypothesis-driven and should not be interpreted as a single linear causal pathway. Created by the authors based on the reviewed literature.

Figure 1 summarizes this model, in which the ECS/endocannabinoidome modulates dopaminergic, GABAergic, glutamatergic, serotonergic, and neuroimmune pathways related to masticatory motor control, stress, sleep, pain, inflammation, and autonomic activity. The model is hypothesis-driven and translational.

### 3.3 Clinical and experimental evidence of cannabinoid use in bruxism

Modulation of the endocannabinoid system (ECS) has been investigated in clinical contexts related to myofascial pain, muscle-related temporomandibular disorders, masseter electromyographic activity, sleep, and bruxism. This literature should be interpreted with caution, since bruxism, temporomandibular disorders, and orofacial pain are not equivalent conditions. However, these phenomena frequently overlap in clinical practice and in the designs of the available studies, since individuals seeking care for pain, muscle tension, or functional impairment often present sleep bruxism, awake bruxism, or both as associated factors. Thus, the existing studies should not be read as direct and homogeneous evidence of therapeutic efficacy for bruxism, but as preliminary clinical evidence, either direct or indirect, on the modulation of outcomes related to masticatory muscle activity, pain, sleep, and function (Nitecka-Buchta et al., 2019; Pina-Escudero et al., 2021; Tanganeli et al., 2023; Pinheiro et al., 2025; Walczyńska-Dragon et al., 2024, 2025a, 2025b).

The clinical trials published to date have focused predominantly on topical or intraoral application of cannabidiol (CBD) in the masseter region. Transdermal CBD application in individuals with myofascial pain related to temporomandibular disorders reduced masseter electromyographic activity and pain intensity, configuring indirect, yet relevant, evidence for the modulation of masticatory muscle activity (Nitecka-Buchta et al., 2019). In individuals with sleep bruxism and muscle-related temporomandibular disorders, a randomized, double-blind, placebo-controlled clinical trial demonstrated reductions in pain, electromyographic activity, and sleep bruxism intensity after CBD intervention (Walczyńska-Dragon et al., 2024). A subsequent clinical publication expanded the evaluation to associated outcomes, including sleep quality and migraine-related disability, in individuals with muscular pain associated with bruxism (Walczyńska-Dragon et al., 2025a). Taken together, these findings suggest that cannabinoid modulation may influence outcomes related to muscle excitability, pain, sleep, and masticatory activity, although they still do not allow definitive therapeutic efficacy to be claimed for sleep bruxism or awake bruxism as independent primary outcomes.

In addition to clinical trials, case reports provide complementary, but lower-level, evidence on the therapeutic potential of phytocannabinoids in complex or refractory clinical presentations. Oral CBD use was associated with marked improvement of awake bruxism in an individual with behavioral variant frontotemporal degeneration, constituting direct evidence for awake bruxism, although limited to a single neurological case (Pina-Escudero et al., 2021). Other case reports involving full-spectrum formulations addressed temporomandibular disorders, orofacial pain, myofascial pain, and sleep dysfunction, contributing indirectly to the clinical rationale, especially in contexts in which chronic pain, muscle dysfunction, and sleep disturbances coexist (Tanganeli et al., 2023; Pinheiro et al., 2025). Although these data do not allow causal inferences, they reinforce the clinical plausibility of ECS modulation in complex phenotypes in which masticatory muscle activity, pain, and sleep-related outcomes overlap.

From a pharmacological perspective, part of the observed effects can be discussed in light of the so-called entourage effect, which describes the synergistic action between cannabinoids, terpenes, and other bioactive compounds present in full-spectrum Cannabis formulations. Experimental evidence and narrative reviews indicate that full-spectrum extracts may promote broader modulation of systems related to motor control, emotional regulation, nociception, and sleep when compared with isolated compounds (Kogan and Mechoulam, 2007; Russo, 2011; Di Marzo and Piscitelli, 2015).

Considering the multifactorial nature of bruxism and the simultaneous participation of motor, emotional, autonomic, nociceptive, and sleep-related circuits, the cross-cutting action of the ECS offers a plausible neurobiological rationale for investigating phytocannabinoid-based strategies. However, the current body of evidence should be interpreted as preliminary and heterogeneous. Larger-scale clinical trials, with longer follow-up, standardized diagnostic criteria, and sleep bruxism and/or awake bruxism as independent primary outcomes are needed to determine the clinical relevance of ECS modulation in bruxism (Nitecka-Buchta et al., 2019; Pina-Escudero et al., 2021; Tanganeli et al., 2023; Pinheiro et al., 2025; Walczyńska-Dragon et al., 2024, 2025a, 2025b). Table 1 presents a critical classification of the available clinical studies, considering study design, condition evaluated, outcomes, direct or indirect relationship with bruxism, and main methodological limitations.

### 3.4 Translational model of ECS-targeted neuromodulation in bruxism

Based on the evidence presented and on previously published therapeutic models for dental management with phytocannabinoids, a translational model for bruxism can be proposed based on modulation of the endocannabinoid system (ECS) (Table 2). This model should be interpreted as a hypothesis-driven construct derived from current neurobiological, clinical, and translational evidence, and not as a universally applicable therapeutic guideline. Table 2 presents titration parameters described for bruxism with and without associated pain, including initial dose, dose increment, route of administration, pharmacological alternatives, and adjunctive strategies (Rosa et al., 2025).

**Table 2:** Hypothesis-driven translational model of cannabinoid-based titration strategies for ECS modulation in bruxism, based on clinical and translational literature.

Clinical context	Reported cannabinoid strategy	Dose ranges reported in literature	Translational considerations
Bruxism without associated pain	CBD-based approach with individualized progressive titration	Initial dose around 15 mg/day of CBD, divided into three daily administrations, with gradual increases of approximately 50% of the previous daily dose every 3 days, according to individual response and tolerability	Focus on modulation of motor excitability and ECS signaling; individualized response variability is expected. Dose should be expressed in milligrams of phytocannabinoid, not only in drops or volume
Bruxism with orofacial pain	CBD-based strategy following the rationale for chronic orofacial pain management	Initial dose around 15 mg/day of CBD, divided into three daily administrations, with progressive titration. If no clinically relevant pain reduction is observed after reaching approximately 40 mg/day of CBD, gradual introduction of Δ9-THC may be considered	Potential effects on nociceptive processing, sleep, stress response and masticatory muscle activity; adjustments should be guided by clinical response, tolerability, functionality and drug interaction profile
Refractory pain associated with bruxism	Combined cannabinoid approaches, including CBD + Δ9-THC or Δ9-THC-containing formulations	Δ9-THC introduced in low doses, such as 1–2.5 mg per dose, divided into three daily administrations, with gradual increases of 1–2.5 mg per dose every 3 days, until pain remission or up to a total daily limit of approximately 40 mg/day	Possible contribution to pain modulation, sleep regulation, reduction of hyperarousal and motor regulation; requires careful monitoring of tolerability, cognition, psychomotor function and drug interactions
CBD or Δ9-THC not feasible or not well tolerated	Alternative cannabinoid profiles, such as CBG-rich formulations	Initial dose around 7.5 mg/day of CBG, divided into three daily administrations, with progressive titration by approximately 50% of the current daily dose every 3 days	Potential neuromodulatory and anxiolytic profile without intoxicating or psychotomimetic effects; clinical evidence remains limited
Adjunctive local management	Topical, transdermal or intraoral CBD-based applications	Variable dosing according to formulation, concentration, route of administration and study protocol; available studies used heterogeneous transdermal, topical or intraoral CBD-based applications	May contribute to local modulation of masseter activity, myofascial pain and sleep bruxism intensity. Heterogeneity of formulations and routes limits direct dose comparison; local, mucosal and systemic contributions remain unclear

The parameters presented in this table represent a hypothesis-driven translational titration model and should not be interpreted as validated dosing recommendations for sleep bruxism or awake bruxism. Individualized assessment, product characterization, dose calculation in milligrams whenever possible, tolerability monitoring and professional follow-up are required. CBD, cannabidiol; CBG, cannabigerol; ECS, endocannabinoid system; Δ9-THC, Δ9-tetrahydrocannabinol.

To date, there is no universally validated dosage for cannabis-derived products in sleep bruxism or awake bruxism. This limitation reflects the scarcity of clinical trials designed to evaluate these phenotypes as independent primary outcomes and the inherent variability of cannabinoid-based therapy, including product composition, concentration, phytocannabinoid ratio, spectrum profile, route of administration, bioavailability, and individual response (MacCallum and Russo, 2018; Rosa et al., 2025).

The principle of “start low, go slow” guides gradual phytocannabinoid titration, beginning with low doses and increasing progressively according to clinical response and tolerability (MacCallum and Russo, 2018; Rosa et al., 2025). In the previously described dental protocol for bruxism without associated pain, the initial strategy consists of administering CBD at a total daily dose of 15 mg, divided into three daily administrations, with progressive adjustment. The suggested increment follows the logic of adding half of the previous daily dose every three days, respecting individual adaptation. After identification of the clinically effective dose, administration may be reorganized into two daily doses when this favors adherence and maintenance of the clinical effect. The dose should be expressed in milligrams of phytocannabinoid, and not only as number of drops or volume, since the concentration per drop varies according to product and manufacturer (Rosa et al., 2025).

In individuals with bruxism associated with orofacial pain, the proposed strategy follows the rationale for chronic orofacial pain management. The protocol begins with CBD at a total daily dose of 15 mg, divided into three daily administrations, with progressive titration. If, after reaching 40 mg/day of CBD, there is no clinically relevant pain reduction, defined as at least 30% reduction, gradual introduction of low-dose Δ9-THC may be considered. In this scenario, the described strategy begins with 1 to 2.5 mg per dose of Δ9-THC, divided into three daily administrations, with an increase of 1 to 2.5 mg per dose every three days, until pain remission or up to the limit of 40 mg/day, always with individual assessment of response, tolerability, functionality, and drug interactions (Rosa et al., 2025).

When the use of CBD or Δ9-THC is not feasible or not well tolerated, cannabigerol (CBG)-rich formulations have been described as an alternative. The proposed strategy begins with 7.5 mg/day of CBG, divided into three daily administrations, with progressive increases of half of the current dose every three days, until reaching the therapeutic target or the limit of individual tolerability (Rosa et al., 2025).

In the available clinical studies, doses, routes of administration, and evaluated outcomes were heterogeneous. In a randomized, double-blind, placebo-controlled clinical trial, a transdermal formulation containing approximately 1.46% CBD was applied to the skin over the masseter region twice daily for 14 days in individuals with myofascial pain related to TMD. Muscle activity was assessed by sEMG in a clinical setting, at rest and during maximum voluntary contraction, on days 0 and 14. The reduction in electromyographic activity indicates lower masseter tension/activity during clinical recording, together with pain reduction. However, since sleep bruxism and awake bruxism were not evaluated as independent primary outcomes, this study constitutes indirect evidence for modulation of masticatory muscle activity related to bruxism (Nitecka-Buchta et al., 2019).

In another randomized, double-blind, placebo-controlled clinical trial, individuals with sleep bruxism and muscle-related TMD were evaluated. CBD formulations at 5% or 10% were applied intraorally to the masseter region before sleep for 30 days. The CBD 5% group received 20 mg/day of CBD, whereas the CBD 10% group received 20 mg per side, totaling 40 mg/day. The study evaluated pain using VAS, muscle tension using sEMG in a clinical setting, and sleep bruxism intensity using Bruxoff during sleep. Reductions were observed in pain, electromyographic activity, and the bruxism index, with more expressive results in the CBD 10% group for pain, muscle tension, and bruxism intensity. The methodological description provided by the authors indicates intraoral application over the masseter region, but does not allow determination of whether the observed effect resulted predominantly from local, mucosal, or systemic absorption (Walczyńska-Dragon et al., 2024).

In a subsequent study, the evaluation was expanded to sleep quality and migraine-related disability in individuals with muscular pain associated with bruxism. Intraoral CBD gel at 5% or 10% was applied directly to the buccal mucosa over the masseter region once daily before sleep for 30 days. Outcomes included PSQI, MIDAS, sEMG, and Bruxoff. Both CBD-treated groups showed significant improvement in sleep quality and migraine-related disability compared with placebo, with no significant difference between the 5 and 10% concentrations for these outcomes. The sEMG findings supported reduced muscle tension, and improvements in sleep and migraine were associated with reduced muscle activity and pain. Thus, this study reinforces the clinical relevance of local CBD modulation in individuals with bruxism associated with muscular pain, but does not establish systemic dosing for bruxism as an isolated outcome (Walczyńska-Dragon et al., 2025a).

Among the case reports, only one directly evaluated bruxism. In that case, almost complete improvement of awake bruxism was reported in an individual with behavioral variant frontotemporal degeneration after morning oral use of a capsule containing 4.8 mg of CBD and 0.31 mg of THC; at the time of the report, bruxism had remained controlled for 6 months (Pina-Escudero et al., 2021). The remaining case reports were included because they evaluated conditions frequently overlapping with bruxism in clinical practice, such as orofacial pain, TMD, muscle dysfunction, and sleep disturbances. In one case of chronic temporomandibular arthralgia, disc displacement with reduction, bilateral degenerative joint disease, and local myalgia, a full-spectrum CBD oil containing 15 mL/900 mg was used. As described by the authors, therapy was initiated with 1 sublingual drop, corresponding to 1.5 mg of CBD, before sleep, with titration up to 2 drops before lunch and 2 drops before sleep, totaling 6 mg/day of CBD, with complete remission of symptoms within 1 week and maintenance of the result at 3- and 6-month follow-up (Tanganeli et al., 2023). In another report, a full-spectrum oil with a 1:1 THC: CBD ratio was used in an individual with chronic muscular TMD, orofacial pain, and sleep dysfunction. The protocol was initiated with 1 sublingual drop twice daily and adjusted every 3 days according to clinical response and tolerability, reaching 5 drops twice daily; however, the dose was reported as number of drops, without conversion into milligrams of THC and CBD, which limits reproducibility and comparison with other studies (Pinheiro et al., 2025). These reports support the plausibility of systemic modulation in complex clinical presentations, but do not allow causal inference or definition of a generalizable dose for bruxism.

In addition to masticatory muscle activity, the translational model considers secondary modulators capable of influencing the frequency, intensity, or persistence of bruxism in specific phenotypes, including anxiety, sleep disturbances, stress response, use of psychostimulants, antidepressants, alcohol, nicotine, and other substances. These factors should not be interpreted as single causes of bruxism, but as elements that may amplify parafunctional masticatory activity or hinder its clinical control. In this context, ECS modulation can be investigated not only for its possible effect on masticatory muscle activity, but also for its potential contribution to the management of associated modulators, including improvement of sleep, reduction of anxiety, control of stress response, and review of medications potentially inducing or exacerbating bruxism, always under professional supervision and with individual risk–benefit assessment (Rosa and Martins, 2024; Conti et al., 2024; Rosa et al., 2025).

Thus, ECS modulation in bruxism should be understood as a translational proposal still under construction. Topical, transdermal, and intraoral CBD formulations provide more direct preliminary clinical evidence on pain, electromyographic activity, and sleep bruxism associated with muscle-related TMD. On the other hand, systemic evidence directly related to bruxism is still restricted to a case report, whereas the remaining systemic case reports contribute indirectly by addressing orofacial pain, TMD, and sleep. Future studies should evaluate sleep bruxism and awake bruxism as primary outcomes, with objective measures, longitudinal follow-up, characterization of formulations, dose reporting in milligrams, and systematic safety monitoring (Nitecka-Buchta et al., 2019; Pina-Escudero et al., 2021; Tanganeli et al., 2023; Pinheiro et al., 2025; Walczyńska-Dragon et al., 2024, 2025a, 2025b; Rosa et al., 2025).

### 3.5 Limitations and future perspectives

Although advances in research on the endocannabinoid system (ECS) have expanded therapeutic possibilities for the management of bruxism, significant scientific and clinical limitations persist that must be considered before widespread adoption of phytocannabinoids in clinical practice. The available body of evidence remains relatively restricted, particularly with regard to large-scale randomized clinical trials with prolonged follow-up.

Much of the clinical research published to date presents small sample sizes, short follow-up duration, and, in some cases, pilot or exploratory design. These methodological characteristics limit the generalization of findings to different population profiles and clinical contexts, particularly regarding the phenotypic variability of bruxism and the coexistence of comorbidities (Nitecka-Buchta et al., 2019; Pina-Escudero et al., 2021; Walczyńska-Dragon et al., 2024). Recent reviews in oral health reinforce that, despite promising results, clinical evidence is still in the initial phase of consolidation, requiring caution in therapeutic extrapolation (Bellocchio et al., 2021, 2023; Viana et al., 2025).

Another limiting aspect concerns the methodological heterogeneity among studies, with wide variation in the formulations employed (isolated cannabinoids versus full-spectrum extracts), ratios of CBD and Δ9-THC, routes of administration (topical, oral, sublingual), treatment duration, and diagnostic criteria used to characterize bruxism. This diversity compromises the standardization of therapeutic protocols and hinders direct comparison between available results, as already highlighted in critical analyses of the cannabinoid literature applied to clinical practice (Iffland and Grotenhermen, 2017; Śmiarowska et al., 2022; Leinen et al., 2023; Maccallum et al., 2023; Duczmal et al., 2024; Rosa and Martins, 2024).

Additionally, there is considerable individual variability in the response to phytocannabinoids, influenced by genetic, metabolic, pharmacokinetic, pharmacological, environmental, and possibly epigenetic factors. This variability requires individualized titration, progressive dose adjustments, and continuous clinical monitoring, especially in medium- and long-term treatments. CBD has an overall favorable tolerability profile and lacks intoxicating effects, although it may be associated with somnolence, sedation, dizziness, fatigue, gastrointestinal symptoms, appetite changes, and drug interactions, especially at higher doses or when used concomitantly with medications metabolized by cytochrome P450 pathways, including CYP3A4, CYP2C9, and CYP2C19. These pathways are relevant for medications such as anticonvulsants, anticoagulants, sedatives, psychotropic drugs, and other long-term medications. Formulations containing Δ9-THC have a distinct profile and require additional caution regarding dose, timing of administration, daytime functionality, xerostomia, cognitive effects, motor coordination, and activities requiring psychomotor attention. In this context, it is important to differentiate potentially therapeutic psychoactive effects, such as relaxation, reduction of hyperarousal, sleep improvement, and modulation of pain perception, from psychotomimetic or dysphoric effects, such as intense anxiety, paranoia, perceptual disorganization, relevant cognitive impairment, or loss of functionality, especially associated with high doses of Δ9-THC, rapid titration, or greater individual susceptibility. Pregnancy, lactation, hypersensitivity to cannabinoids or excipients, relevant liver disease, glaucoma or ocular hypertension, and concomitant use of medications with higher interaction risk should be evaluated before prescribing any cannabinoid formulation. In individuals with Sjögren’s syndrome or marked hyposalivation/xerostomia, history of psychosis, severe non-stabilized psychiatric disorders, greater vulnerability to psychotomimetic effects, or professional activities requiring full psychomotor attention, caution is particularly relevant when Δ9-THC-containing formulations are considered. These aspects reinforce the need for prior clinical assessment, review of current medications, individualized guidance, control of product quality and composition, and longitudinal monitoring of clinical response and tolerability. Pharmacological and clinical reviews emphasize that this variability is intrinsic to cannabinoid-based therapy and to the action of the ECS as a homeostatic system, requiring specific training of the prescribing professional and individualized monitoring (Iffland and Grotenhermen, 2017; MacCallum and Russo, 2018; Śmiarowska et al., 2022; MacCallum et al., 2023; Rosa et al., 2025).

From a regulatory perspective, the therapeutic use of Cannabis in dentistry still faces legal and institutional barriers in various countries. In the Brazilian context, although dental prescription of phytocannabinoids is permitted through professional qualification, challenges persist related to product regulation, quality control, traceability, standardization of formulations, and health surveillance. The absence of specific clinical guidelines for dentistry contributes to professional insecurity and limits the systematic incorporation of this approach, despite consistent growth in scientific production in the field (Bellocchio et al., 2021, 2023; Maccallum et al., 2023; Viana et al., 2025).

Another critical point concerns the scarcity of studies that systematically evaluate the integration of phytocannabinoids with other therapeutic modalities frequently employed in the management of bruxism, such as orofacial physiotherapy, cognitive-behavioral therapy, occlusal rehabilitation, and judicious use of interocclusal devices. Investigation of combined approaches represents a promising perspective for enhancing clinical efficacy, reducing treatment time, and increasing individual adherence to proposed therapeutic protocols (Manfredini et al., 2020; Conti et al., 2024; Rosa and Martins, 2024).

Despite these limitations, the pathophysiological rationale supporting the use of phytocannabinoids in bruxism remains consistent. The condition is associated with dysregulation of the dopaminergic, GABAergic, serotonergic, and glutamatergic pathways, which are modulated in an integrated manner by the ECS. This system acts transversally over central circuits involved in motor control, stress response, pain, and autonomic regulation, offering a plausible neurobiological basis for its therapeutic application in conditions of central origin with peripheral muscular manifestations, such as bruxism (Di Marzo and Piscitelli, 2015; Covey et al., 2017; Arturo and Fabiana, 2018; Di Marzo, 2018; Iannotti and Di Marzo, 2025).

For the proposed model to be tested in evidence-based clinical contexts, randomized, placebo-controlled clinical trials with prospective design, longitudinal follow-up, and predefined sleep bruxism and/or awake bruxism as primary outcomes are needed. In sleep bruxism, future studies should prioritize objective measures of masticatory muscle activity, such as surface electromyography, polysomnography, or validated ambulatory recording devices, associated with the evaluation of sleep parameters. In awake bruxism, ecological momentary assessment may be used to measure the frequency, intensity, and context of clenching or tooth-contact episodes during the day. Secondary outcomes should include orofacial pain, mandibular function, sleep quality, anxiety, stress, quality of life, concomitant medication use, and adverse events. In addition, interventions should be described with product characterization, composition, concentration, route of administration, titration, dose expressed in milligrams of phytocannabinoids, treatment duration, and monitoring of tolerability and safety. When possible, the inclusion of markers related to the ECS/endocannabinoidome, inflammation, stress response, or sleep architecture may contribute to testing the biological mechanisms underlying the proposed translational model (Nitecka-Buchta et al., 2019; Bellocchio et al., 2023; Walczyńska-Dragon et al., 2024, 2025b; Rosa et al., 2025).

Finally, the importance of developing specific clinical guidelines for dentistry is emphasized, developed with the participation of regulatory entities, professional associations, and researchers in the field. The creation of interdisciplinary centers for teaching, research, and care in cannabinoids in dentistry can accelerate the consolidation of this integrative approach, promoting greater safety, efficacy, and humanization in the care of individuals with bruxism (Bellocchio et al., 2021, 2023; Rosa and Martins, 2024; Viana et al., 2025).

## 4 Conclusion

For decades, bruxism was understood as a condition requiring predominantly peripheral management, with almost exclusive focus on palliative strategies aimed at containing or minimizing the mechanical effects of parafunctional muscle activity. However, advances in neurobiological knowledge have consolidated the understanding of bruxism as a centrally mediated motor behavior, associated with the modulation of dopaminergic, serotonergic, GABAergic, and glutamatergic pathways, which are fundamental for motor control, emotional regulation, and stress response. These pathways are under direct influence of the endocannabinoid system (ECS), recognized as a cross-cutting homeostatic modulator of excitatory and inhibitory neurotransmission in the central nervous system (Di Marzo and Piscitelli, 2015; Covey et al., 2017; Di Marzo, 2018).

In this context, modulation of the ECS through phytocannabinoids represents a biologically plausible hypothesis for influencing central processes involved in bruxism. The action of cannabinoids on neural circuits related to pain, muscle tone, anxiety, stress, and sleep regulation suggests a potential role in modulating the multidimensional processes underlying the clinical expression of bruxism, both during sleep and wakefulness (Kogan and Mechoulam, 2007; Di Marzo, 2018).

The clinical evidence currently available, although still limited in number, supports this neurobiological rationale. Randomized controlled clinical trials have demonstrated that topical use of cannabidiol may reduce orofacial myofascial pain and electromyographic activity of the masticatory muscles, and may influence sleep bruxism intensity in selected clinical contexts, with a favorable safety profile and good tolerability (Nitecka-Buchta et al., 2019; Walczyńska-Dragon et al., 2024, 2025a, 2025b). These findings support the relevance of peripheral and central ECS modulation as a potential mechanism influencing muscle hyperactivity associated with bruxism, but should not be interpreted as definitive evidence of efficacy for sleep bruxism or awake bruxism as independent primary outcomes.

Additionally, case reports involving systemic cannabinoid-based formulations provide complementary clinical plausibility in complex or refractory presentations, including awake bruxism, orofacial pain, temporomandibular disorders, muscle dysfunction, and sleep disturbances (Pina-Escudero et al., 2021; Tanganeli et al., 2023; Pinheiro et al., 2025). However, this level of evidence remains limited by the small number of cases, absence of control groups, heterogeneous formulations, and incomplete dose standardization in some reports.

From a pharmacological perspective, full-spectrum extracts may be discussed through the concept of the entourage effect, according to which the synergistic interaction between cannabinoids, terpenes, and other phytocompounds may result in broader modulation of neurochemical systems involved in pain, anxiety, sleep, and motor control (Russo, 2011; Di Marzo and Piscitelli, 2015).

Despite these advances, the full incorporation of phytocannabinoids into clinical practice still requires caution. The literature lacks multicentric clinical trials with larger samples, longitudinal follow-up, and standardized objective outcomes capable of defining with greater precision the indications, dosing strategies, routes of administration, and safety profiles for bruxism. Future studies should evaluate sleep bruxism and awake bruxism as predefined primary outcomes, use objective and ecological measures of masticatory muscle activity, characterize formulations and doses in milligrams, and systematically monitor safety, tolerability, and drug interactions. Furthermore, regulatory, educational, and institutional challenges continue to limit the safe and systematic integration of this approach (Nitecka-Buchta et al., 2019; Bellocchio et al., 2023; Rosa and Martins, 2024; Walczyńska-Dragon et al., 2024, 2025b).

In summary, the integration of neurobiological knowledge about bruxism with advances in understanding the endocannabinoid system supports the development of a hypothesis-driven translational model in which ECS-mediated neuromodulation may represent a relevant pathway for influencing centrally mediated motor behaviors. By recognizing bruxism as an expression of complex neuromodulatory dysregulation, this framework provides a conceptual basis for future mechanistic and clinical investigation, rather than a prescriptive therapeutic guideline.

## Data Availability

The original contributions presented in the study are included in the article/supplementary material, further inquiries can be directed to the corresponding author.
